# Autoantibodies Recognizing Secondary NEcrotic Cells Promote Neutrophilic Phagocytosis and Identify Patients With Systemic Lupus Erythematosus

**DOI:** 10.3389/fimmu.2018.00989

**Published:** 2018-05-07

**Authors:** Mona H. C. Biermann, Sebastian Boeltz, Elmar Pieterse, Jasmin Knopf, Jürgen Rech, Rostyslav Bilyy, Johan van der Vlag, Angela Tincani, Jörg H. W. Distler, Gerhard Krönke, Georg Andreas Schett, Martin Herrmann, Luis E. Muñoz

**Affiliations:** ^1^Department of Internal Medicine 3 – Rheumatology and Immunology, Friedrich-Alexander-Universität Erlangen-Nürnberg (FAU) and Universitätsklinikum Erlangen, Erlangen, Germany; ^2^Department of Nephrology, Radboud University Medical Centre, Nijmegen, Netherlands; ^3^Danylo Halytsky Lviv National Medical University, Lviv, Ukraine; ^4^Division of Rheumatology and Clinical Immunology, Department of Clinical and Experimental Sciences, Spedali Civili and University of Brescia, Brescia, Italy

**Keywords:** systemic lupus erythematosus, secondary necrosis, autoimmunity, autoantibodies, inflammation, connective tissue diseases

## Abstract

Deficient clearance of apoptotic cells reportedly contributes to the etiopathogenesis of the autoimmune disease systemic lupus erythematosus (SLE). Based on this knowledge, we developed a highly specific and sensitive test for the detection of SLE autoantibodies (AAb) utilizing secondary NEcrotic cell (SNEC)-derived material as a substrate. The goal of the present study was to validate the use of SNEC as an appropriate antigen for the diagnosis of SLE in large cohort of patients. We confirmed the presence of apoptotically modified autoantigens on SNEC (dsDNA, high mobility group box 1 protein, apoptosis-associated chromatin modifications, e.g., histones H3-K27-me3; H2A/H4 AcK8,12,16; and H2B-AcK12). Anti-SNEC AAb were measured in the serum of 155 patients with SLE, 89 normal healthy donors (NHD), and 169 patients with other autoimmune connective tissue diseases employing SNEC-based indirect enzyme-linked immunosorbent assay (SNEC ELISA). We compared the test performance of SNEC ELISA with the routine diagnostic tests dsDNA Farr radioimmunoassay (RIA) and nucleosome-based ELISA (*anti-dsDNA-NcX-ELISA*). SNEC ELISA distinguished patients with SLE with a specificity of 98.9% and a sensitivity of 70.6% from NHD clearly surpassing RIA and *anti-dsDNA-NcX-ELISA*. In contrast to the other tests, SNEC ELISA significantly discriminated patients with SLE from patients with rheumatoid arthritis, primary anti-phospholipid syndrome, spondyloarthropathy, psoriatic arthritis, and systemic sclerosis. A positive test result in SNEC ELISA significantly correlated with serological variables and reflected the uptake of opsonized SNEC by neutrophils. This stresses the relevance of SNECs in the pathogenesis of SLE. We conclude that SNEC ELISA allows for the sensitive detection of pathologically relevant AAb, enabling its diagnostic usage. A positive SNEC test reflects the opsonization of cell remnants by AAb, the neutrophil recruitment to tissues, and the enhancement of local and systemic inflammatory responses.

## Introduction

Systemic lupus erythematosus (SLE) is a chronic inflammatory autoimmune disease. Its pathogenesis is multifactorial including the involvement of genetic (1), hormonal (2), immunologic (3), and environmental factors like infections (4, 5). The appearance of autoantibodies (AAb) is the hallmark of systemic autoimmunity in SLE and results from a series of immune-mediated events that typically precede the onset of clinical symptoms by several years. The development of autoimmunity is considered to be partly triggered by impaired clearance of apoptotic cells in the germinal centers of the lymph nodes (6–9). Apoptotic cells that are not cleared rapidly by professional phagocytes lose their membrane integrity and, consequently, release cytoplasmic and nuclear autoantigens. These antigens are usually connected to damage-associated molecular patterns (DAMPs) like the high mobility group box 1 protein (HMGB1) (10). The material generated in the absence of a proper clearance is designated as secondary NEcrotic cells (SNECs) (11–13). The concomitant release of DAMPs and nucleic acids triggers an inflammatory response (14, 15) which, in combination with the accessibility of autoantigens, precipitates the production of SNEC-specific AAb (16). After autoimmunity is established uncleared post-apoptotic material serves as autoantigen repository for the formation of pathogenic immune complexes (ICs) (17, 18). These complexes form *in situ* or deposit in various tissues, especially in the kidney, skin, and joints, where they trigger inflammation and tissue damage (19, 20).

In 1948, Hargraves discovered the LE cell as first test for diagnosing SLE [reviewed in Ref. (21)] representing a phagocytic cell that has ingested the secondary necrotic nucleus of another cell closely resembling SNEC (22). AAb against nuclear proteins are essential to form LE cells (23, 24) suggesting recognition of SNEC in the context of autoimmunity in SLE (25, 26). Accordingly, LE cells reportedly indicate serologically and clinically active disease with major organ involvement. After several decades, the LE cell test was replaced by serum autoantibody testing in 1997, not least because LE cell testing is time consuming and challenging (27, 28). The presence of AAb increases the risk for clinical disease by at least 40-fold (29). A plethora of autoantibody specificities can be detected in patients with SLE that comprise reactivities against dsDNA, nucleosomes, RNA-protein complexes, Smith antigen (Sm), and ribosomal proteins (30).

Considering the aforementioned pathophysiologic events, we hypothesized that the detection of anti-SNEC AAb is a highly specific and potentially sensitive tool for the classification of SLE. Thus, the goal of the present study was to validate the use of SNEC as an appropriate antigen for the diagnosis of SLE in large cohort of patients. Employing SNEC as antigen, we developed a specific and sensitive high-throughput test to identify patients with pathogenic AAb against post-apoptotic cells. This anti-SNEC enzyme-linked immunosorbent assay (ELISA) discriminated SLE patients from healthy individuals and patients with other autoimmune connective tissue diseases with a specificity and sensitivity of 98.9 and 70.6%, respectively, surpassing currently used standard detection methods.

## Materials and Methods

### Patient and Normal Healthy Donor (NHD) Serum Samples

This study was carried out in accordance with the recommendations of institutional guidelines and the approval of the ethical committee of the Universitätsklinikum Erlangen (permit # 54_14B). The protocol was approved by the ethical committee of the Universitätsklinikum Erlangen (permit # 54_14B). Written informed consent was given by each donor in accordance with the Declaration of Helsinki. Serum samples from NHD and patients with SLE, RA, SpA, PsA, and SSc, fulfilling the 1997 American College of Rheumatology criteria, were obtained at the Department of Rheumatology and Immunology of the Universitätsklinikum Erlangen. Sera from patients with primary anti-phospholipid syndrome (PAPS) were obtained from the Department Rheumatology and Clinical Immunology of the Spedali Civili and University of Brescia. Samples were stored at −20°C until analysis.

### Preparation of SNECs

Peripheral blood mononuclear cells (PMBC) were obtained from heparinized whole NHD blood and isolated by density gradient-based isolation using Lymphoflot (Bio-Rad, Dreieich, Germany) as previously described (31). Isolated PBMCs were adjusted to a concentration of 5 × 10^6^ cells/ml in PBS and irradiated using 240 mJ/cm^2^ UVB light for 90 s. After incubation for 24 h at 37°C and antigen retrieval at 56°C, SNEC was stored at −20°C containing 5 mM EDTA. Before coating, SNEC was washed in 10 mM Tris buffer containing 1 mM EDTA (pH 8.0). For *ex vivo* phagocytosis assays, SNEC was concentrated to 15 × 10^7^ cells/ml and labeled with propidium iodide (PI).

### *Ex Vivo* Phagocytosis Assays

Fresh heparinized whole blood from NHD was added to polystyrene tubes and 12% serum of NHD or patients with SLE and 10% PI-stained SNEC (15 × 10^7^/ml) was added. Samples were incubated for 4 h at 37°C to allow uptake of SNEC by phagocytes and stained for HLA-DR (FITC), CD16 (APC), and DNA (Hoechst33342) for 30 min at 4°C in the dark. After hypotonic lysis of erythrocytes and fixation of the cells, samples were measured by flow cytometry (Gallios™ Beckman Colter, Krefeld, Germany) and analyzed using Kaluza 1.5 software (Beckmann Colter). Uptake of SNEC is presented as *phagocytosis index* calculated using the percentage of PI-positive cells and the mean fluorescent intensity.

### SNEC ELISA

The serum of NHD and patients affected by several pathological conditions was analyzed by ELISA for the presence of anti-SNEC IgG AAb. 96-well microtiter plates (Nunc-Immuno™Maxisorp) were coated overnight at 4°C with 50 µl of Poly-l-Lysine (20 µg/ml) in 10 mM Tris buffer containing 1 mM EDTA (pH 8.0). Plates were washed three times with 200 µl of washing buffer [phosphate-buffered saline (PBS), 0.05% Tween 20, pH 7.4] after each incubation step. After coating overnight at 4°C with SNEC in 10 mM Tris buffer containing 1 mM EDTA (pH 8.0), plates were blocked for 1 h at room temperature (RT) with 100 µl blocking agent (2% BSA in PBS) per well, 50 µl of serum per well (1:200 in PBS-T) was added and incubated for 1 h at RT. Next, 50 µl of goat anti-human IgG Fc HRP-conjugated (Southern Biotech, Birmingham, AL, USA) antibody (1:50,000 in washing buffer) was added and incubated for 1 h at RT. Finally, plates were incubated with 50 µl of substrate solution [substrate buffer (0.1 M Na_2_HPO_4_, 0.05 M citrate acid, pH 5.0), 10% TMB, and 0.02% H_2_O_2_ (30%)] for 5 to 10 min. The reaction was stopped with 50 µl of H_2_SO_4_ (25%) and absorbances were read at 450 and 620 nm reference wavelength. To account for differences between plates, values were corrected using a standard serum applied on each plate.

### Characterization of SNEC Autoantigens by ELISA

ELISA was performed as described above. Antibodies detecting several autoantigens [mAb #34: mouse anti-histone H3 (32); mAb #42: mouse anti-DNA; mAb BT131: mouse anti-apoptotic nucleosome (33); mAb BT164: mouse anti-H3-K27me3 (34); mAb KM-2: mouse anti-H4-AcK8,12,16 (35); mAb LG11-2: mouse anti-H2B-AcK12 (36); rabbit anti-HMGB1 (Abcam, Cambridge, UK)] were added at the indicated concentrations in PBS-T (containing 1% BSA) and incubated for 1 h at RT. The respective secondary antibodies, goat anti-mouse IgG Fc HRP-conjugated (Jackson ImmunoResearch, Suffolk, UK), goat anti-rabbit IgG Fc HRP-conjugated (Southern Biotech, Birmingham, AL, USA), and goat anti-human IgG Fc HRP-conjugated (Southern Biotech, Birmingham, AL, USA) were added (1:10,000 in PBS-T, 1% BSA) for 1 h at RT.

### Immunofluorescence Staining

Eight-well Nunc chamber slides (VWR, Darmstadt, Germany) were coated, washed, and blocked as described above. Wells were incubated with the serum of one NHD with a low SNEC ELISA value and the serum of one SLE patient with a high SNEC ELISA value (1:200 in PBS-T) or antibodies detecting histone H3 (mAb #34) and mouse anti-H3-K27me3 (mAb BT164). Goat anti-human IgG FITC (Jackson ImmunoResearch, Suffolk, UK), goat anti-mouse IgG Cy^®^5 (Jackson ImmunoResearch, Suffolk, UK), and goat anti-rabbit IgG Cy^®^5 (Jackson ImmunoResearch, Suffolk, UK) were added and incubated for 1 h in the dark. A control staining lacking a primary antibody was included. Finally, slides were washed with PBS and H_2_O, embedded in DAKO fluorescent mounting medium (Agilent Technologies, Santa Clara, CA, USA) and analyzed using the Eclipse Ni-U (Nikon Corporation, Tokyo, Japan).

### Live Fluorescence Microscopy

Fresh heparinized whole blood from NHD was obtained and 12% serum of patients with SLE and 10% PI-stained SNEC (150 × 10^6^ cells/ml) were added. The samples were incubated for 1 h at 37°C. After hypotonic lysis of erythrocytes and fixation with 4% PFA, cells were deposited on chamber slides. Slides were blocked with 10% FCS and stained for CD15 (APC) and DNA (Hoechst33342) and the respective isotype control antibody for 2 h at RT in the dark and analyzed using the Eclipse Ni-U.

### Adsorption of Anti-SNEC AAb From SLE Serum

Serum C-reactive protein (CRP) was purified using immobilized p-Aminophenyl Phosphoryl Choline agarose gel (Thermofisher) as per the manufacturer’s instructions. CRP bound to agarose was incubated with SNEC for 1 h at RT (13). Anti-SNEC AAb were removed from SLE serum by incubation with SNEC-bound CRP agarose by incubation for 1 h at RT.

### Statistical Data Analysis

Age, sex, comorbidities, organ involvement, and disease onset were recorded for all patients. CRP levels, erythrocyte sedimentation rate (ESR), rheumatoid factor (RF), dsDNA radioimmunoassay (RIA), anti-dsDNA-NcX-ELISA, extractable nuclear antigen (ENA) profiles, and complement levels (C3 and C4) were measured on the same day of serum collection. Disease activity score European Consensus Lupus Activity Measurement Manifestation (ECLAM) was calculated. For comparisons between two groups, Mann–Whitney *U*-tests for numerical variables and exact χ^2^-tests for nominal characteristics were employed. ANOVA with Bonferroni *Post Hoc* correction was employed for comparisons among several groups. Associations among clinical and laboratory variables were measured by Bravais-Pearson correlation coefficients and corrected after Bonferroni. The discriminatory power of a test was evaluating calculating the receiver operating characteristic (ROC) method. Cumulative predicted probabilities [the area under the curve, positive predictive value (PPV), negative predictive value (NPV), diagnostic odds ratio (DOR), cutoff, sensitivity, specificity] were calculated for numeric comparison of ROC curves. All statistical analyzes were performed using IBM SPSS Software (version 21) and GraphPad Prism 5.03 software. A *p* value ≤0.05 was considered statistically significant.

## Results

### AAb From Patients With SLE Recognize SNEC-Borne Autoantigens Modified During Apoptosis

**Figure 1:**
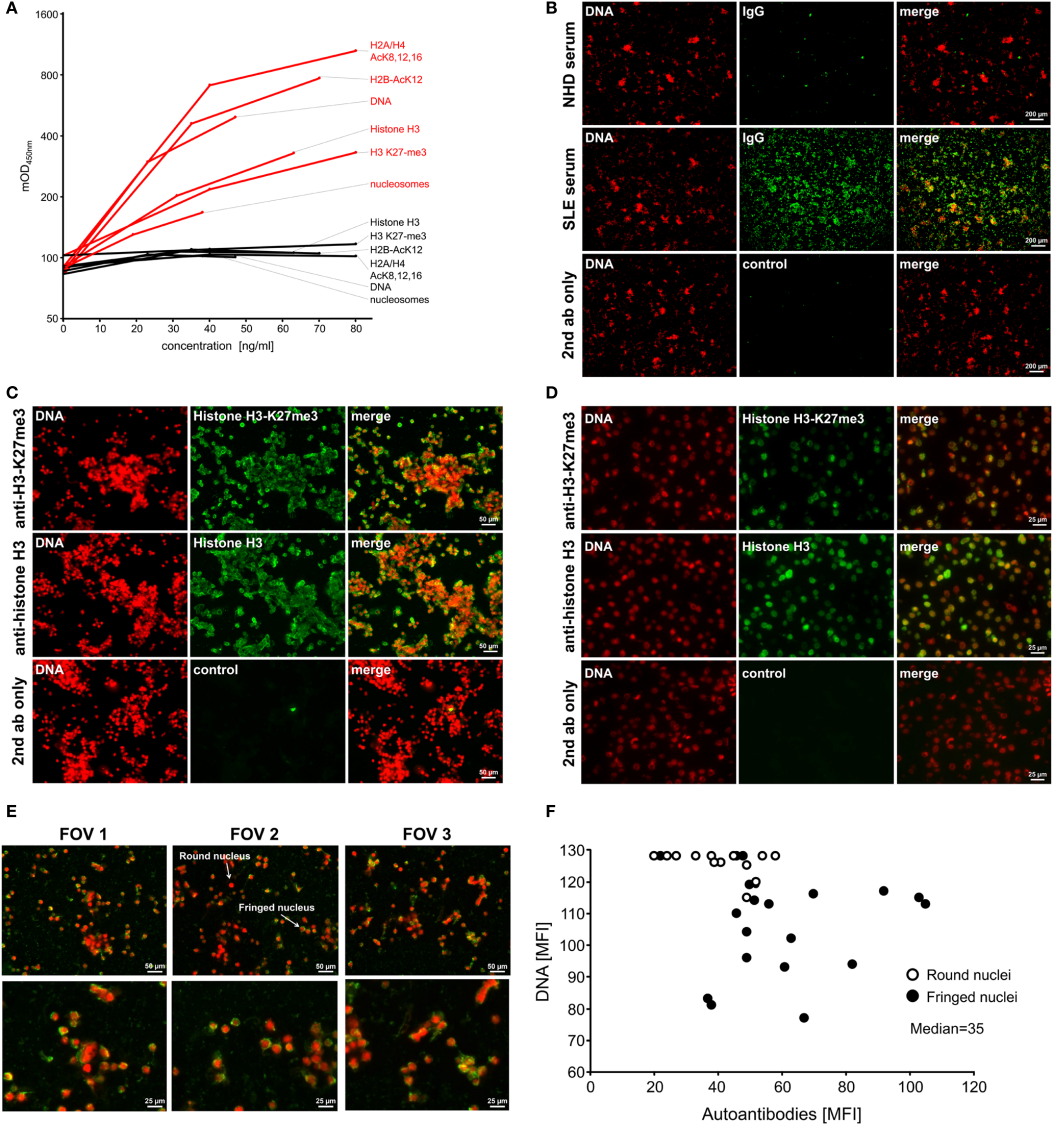
Immobilized secondary NEcrotic cells (SNECs) expose naïve and modified nuclear autoantigens. **(A)** Recognition of immobilized autoantigens on SNEC by specific antibodies. Coated material contained histone H3 (#34), DNA (#42), nucleosomes (BT131), and the apoptotically modified histones H3 K27-me3 (BT164), H2A/H4 AcK8,12,16, (KM-2), and H2B-AcK15 (LG11-2). Respective isotype control antibodies (iso) displayed background signals. **(B)** Immunofluorescence of SLE-borne IgG autoantibodies (AAb) binding immobilized SNEC counter-stained for DNA by propidium iodide (PI). NHD, normal healthy donor; SLE, systemic lupus erythematosus; magnification 20×. **(C)** Immunofluorescence of histone H3 and modified histone H3 K27-me3 on immobilized SNEC counter-stained for DNA by PI; magnification 40×. **(D)** Immunofluorescence staining of histone H3 and modified histone H3 K27-me3 on HEp-2 cells. **(E)** Immunofluorescence of binding pattern of IgG autoantibodies present in the serum of patients with SLE to immobilized SNEC stained for DNA by PI; FOV, field of view. **(F)** Morphometric evaluation of **(E)**, intensity of DNA staining versus the intensity of IgG staining.

We employed SNEC as immobilized antigen in an indirect ELISA (Figure S1A in Supplementary Material) to detect SLE-specific antibodies. We demonstrated the availability of specific apoptotically modified nuclear autoantigens on SNEC (Figure 1A) and confirmed the presence of DNA, histone H3, nucleosomes, and histones carrying several apoptotic modifications (histone H3 K27-me3; histone H2A/H4 AcK8,12,16, histone H2B-AcK12). Representative pictures of repeated SNEC antigen characterization are shown. Analyzes by immunofluorescence revealed that autoreactive IgG present in the sera of patients with SLE strongly bound to immobilized SNEC, whereas sera of NHD lacked reactivity (Figure 1B). SNEC was recognized by DNA- and nucleosome-specific AAb in a particular manner (Figure 1C) resembling the recognition pattern of SLE serum-derived AAb (Figure 1C). These monoclonal antibodies also recognized antigens on HEp-2 cells; however, apoptotically modified histone H3 was recognized to a lesser extent (Figure 1D). HMGB1 was also found on immobilized SNEC linked to apoptotic DNA (Figure S1B in Supplementary Material). Murine AAb bound SNEC in a unique pattern preferentially targeting cells with low DNA densities (Figure 1C). Morphometry confirmed that human SLE AAb showed a similar binding pattern preferring cells with fringed nuclei and decondensed chromatin when compared to targets with bright round nuclei (Figures 1E,F).

### The Test Performance of SNEC ELISA Surpasses That of Current Routine Assays

To establish a reproducible assay, we tested the linearity of SNEC ELISA employing serial dilutions of positive and negative sera (Figure S1C in Supplementary Material). For further analyzes, we used a serum dilution factor of 200 lying in the linear detection range. Comparison of different apoptosis stimuli resulted in comparable autoantibody detection for UVB- or Dexamethasone-induced apoptosis (Figure S1D in Supplementary Material). To assess the performance of SNEC ELISA, we studied 155 patients with SLE [median age 44.5 years (inter quartile range (IQR) 22–42)] and 89 normal healthy donors (NHD) [median age 27.5 years (IQR 25–33)] (Table 1). A summary of demographical, serological, and clinical data of all study cohorts is presented in Tables 1 and 2. The reproducibility of SNEC ELISA in terms of batch variability was tested and corrected using a standard serum, which revealed reproducible signals.

**Table 1:** Demographical and serological characteristics of the study cohorts.

		NHD	SLE	PAPS	RA	SpA	PsA	SSc
*n*		89	155	37	53	20	30	29
Sex	(Female/male)	52/33	133/22	28/9	40/13	7/13	14/16	19/9
Current age (years)	Median/IQR	27.5/25–33	44.5/22–42	47/37–40	62.8/53–71	46.2/36–57	54.1/46–63	55.3/50–72
Age at diagnosis (years)	Median/IQR	na	29.2/10–76	na	45.3/36–57	31.6/18.5–41.6	40.2/33–53	47.9/42–62
CRP (mg/dl) [<5 mg/L]	Median/IQR	na	3.7/2.2–6.2	na	3.5/2.3–8.3	4.9/2.55–9.5	5.0/2.1–7.0	6.3/2.8–9.4
RF (IE/ml) [0–20 IE/ml]	Median/IQR	na	na	na	25.5/0–183	na	na	na
ACPA (U/ml) [<10 U/ml]	Median/IQR	na	na	na	57.5/3.6–294.3	na	na	na
a-SNEC IgG (mOD) [<246 mOD]	Median/IQR	85.0/64.0–121.5	309.8/239.4–375.3	61.0/12.4–94.0	85.9/42.0–148.8	50.6/38.5–86.4	52.0/34.6–92.9	109.0/89.9–122.1
SNEC ELISA positivity	%	1.1	70.6	0.0	1.9	0.0	0.0	3.4

*ACPA, anti-citrullinated peptide antibody; a-SNEC IgG, anti-secondary necrotic cell-IgG antibody; CRP, C-reactive Protein; IE, Internationale Einheit (engl. International units); IQR, interquartile range; mOD, mean optical density; na, not available; PAPS, primary anti-phospholipid syndrome; PsA, psoriatic arthritis; RA, rheumatoid arthritis; RF, rheumatoid factor; SLE, systemic lupus erythematosus; SpA, spondyloarthropathy; SSc, systemic sclerosis; U, unit; NHD, normal healthy donor*.

**Table 2:** Serological and clinical characteristics of the SLE cohort.

	Variable	*n*	Mean ± SD	Frequency altered (%)
Serology	ANA on HEp-2 (1/) [≥1/320]	152	1544.2 ± 3389.3	71.2
	Radioimmunoassay (RIA) dsDNA (U/ml) [0–7 U/ml]	153	22.3 ± 79.2	39
	C3 (mg/dl) [81.1–157 mg/dl]	153	87.6 ± 21.3	63
	C4 (mg/dl) [12.9–39.2 mg/dl]	153	15.4 ± 5.7	14
	CRP (mg/l) [<5 mg/L]	152	5.4 ± 6.20	17
	Anti-CL IgG [0–14 GPL U/ml]	56	28.0 ± 56.2	37.5
	Anti-β2GP IgG [0–10 U/ml]	56	12.3 ± 25.3	21.4
	ESR (mm/60min) [<20 *F*; <15 M mm/60 min]	146	18.7 ± 13.5	42
	a-SNEC IgG [mean optical density (mOD)] [<246 mOD]	153	264.5 ± 100.6	71
	Ncx dsDNA ELISA (IE/ml) [<100 IE/ml]	153	146.9 ± 187.6	43

ENA [0–3]	Histones	155	0.72 ± 1.05	41
	Nucleosomes	155	0.74 ± 1.06	44
	Ro-52 (52 kDa)	155	0.85 ± 1.29	36
	SS-A (60 kDa)	155	0.99 ± 1.34	39
	RNP/Sm	155	0.71 ± 1.21	31
	AMA-M2	155	0.15 ± 0.54	11
	Ribosomal P protein	155	0.21 ± 0.57	17
	PCNA	155	0.05 ± 0.20	6
	Centromer B	155	0.04 ± 0.24	4
	Jo-1	155	0.03 ± 0.20	3
	PM-Scl	155	0.08 ± 0.26	13
	Scl-70	155	0.07 ± 0.36	5
	SS-B	155	0.25 ± 0.78	12
	RNP C	155	0.09 ± 0.37	9
	RNP A	155	0.31 ± 0.83	15
	RNP 70	155	0.25 ± 0.73	12
	Sm	155	0.20 ± 0.64	12

Clinic	SLEDAI (0–105)	84	2.82 ± 2.65	
	ECLAM (1–10)	153	5.73 ± 2.51	

*C3/C4; complement factor 3/4; Anti-β2GP1 IgG, anti-β2 glycoprotein1 IgG; anti-CL, anti-cardiolipin IgG; ANA, anti-nuclear antigen; ENA, extractable nuclear antigen; CRP, C-reactive protein; ESR, erythrocyte sedimentation rate; ECLAM, European Consensus Lupus Activity Measurement Manifestation; SLEDAI, SLE disease activity index*.

**Figure 2:**
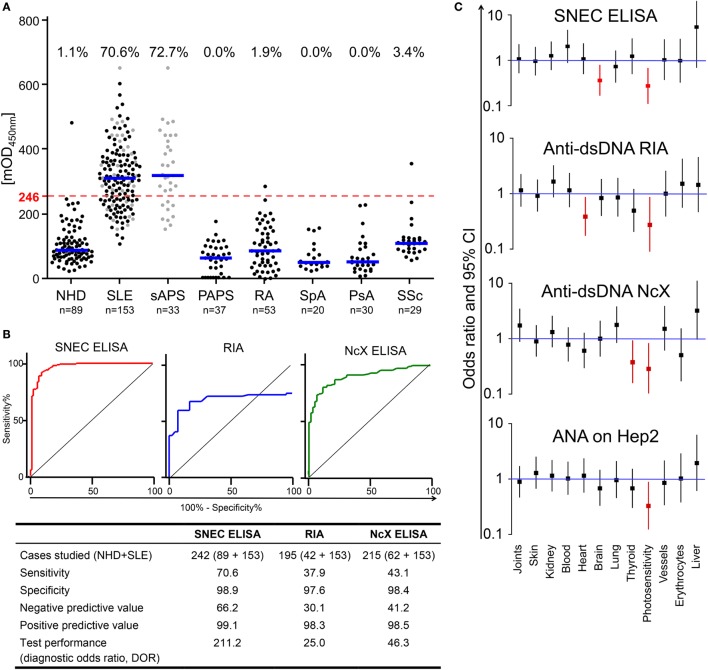
SLE-derived AAb are specifically and sensitively detected by secondary NEcrotic cell (SNEC) ELISA. **(A)** Reactivity of sera from normal healthy donors (NHD) and patients with SLE, secondary anti-phospholipid syndrome (sAPS, gray dots in the SLE cohort), primary anti-phospholipid syndrome (PAPS), spondyloarthropathy (SpA), psoriatic arthritis (PsA), and systemic sclerosis (SSc) against SNEC were assessed by ELISA. Using receiver operating characteristic (ROC) analysis, the cut-off for positive values was calculated at 246 mean optical density (dashed red line). Frequencies of SNEC positivity are displayed above the respective cohort in percent (%). **(B)** The overall test performance of the SNEC ELISA (left), radioimmunoassay (RIA) dsDNA Farr assay (center), and anti-dsDNA-NcX-ELISA (right) was evaluated employing ROC analysis. The respective diagnostic parameters (specificity, sensitivity, negative predictive value, positive predictive value, and diagnostic odds ratio) were calculated and are displayed below. **(C)** Organ involvement was analyzed by comparison of odds ratios for SNEC ELISA, anti-dsDNA RIA, anti-dsDNA-NcX ELISA, and anti-nuclear antigen on HEp-2 titers. The depicted odd ratios are within the 95% confidence interval.

Secondary NEcrotic cell ELISA values from individuals of the NHD and SLE cohorts were not dependent on age (Figures S1F,G in Supplementary Material). ROC analyzes using the NHD sera as control cohort determined positive sera above the cutoff of 246 mOD (Figure 2A). 70.6% of patients with SLE showed seropositivity in SNEC ELISA, whereas just 1.1% of NHD were positive (Figure 2A). Patients with secondary anti-phospholipid syndrome (sAPS, 72.7%) are depicted additionally in a separate cohort and are undistinguishable from the patients with SLE without sAPS (Figure 2A). We employed sera from patients with other connective tissue disorders, namely PAPS (0%), rheumatoid arthritis (RA, 1.9%), spondyloarthropathy (SpA, 0%), psoriatic arthritis (PsA, 0%), or systemic sclerosis (SSc, 3.4%) (Figure 2A) showing cumulatively only 1.2% seropositivity in SNEC ELISA (Figure 2A).

Receiver operating characteristic analysis showed a specificity of 98.9% at a sensitivity of 70.6% for SNEC ELISA (Figure 2B) surpassing current routine assays like the *anti-dsDNA-NcX-ELISA* (EUROIMMUN) and the Farr radioimmunoassay (IBL International) in our cohort (Figure 2B). Analysis of the ROC curves of combined results of RIA and ENA nucleosome or ENA histone revealed that the sensitivity of SNEC ELISA also outperformed the combination of these tests (Figure S1E and Table S1 in Supplementary Material). A PPV of 99.1% for SNEC ELISA confirmed the high accuracy of the assay (Figure 2B), comparable to RIA and *anti-dsDNA-NcX-ELISA* (98.3 and 98.5%, respectively). The NPV, indicating the percentage of true negative test results, was 66.2% for SLE, clearly exceeding RIA and *anti-dsDNA-NcX-ELISA* (NPV: 30.1 and 41.2%). SNEC ELISA was the most effective test with a DOR of 211.2, performing better than RIA (DOR 25.0) and *anti-dsDNA-NcX-ELISA* (DOR 46.3) (Figure 2B).

**Figure 3:**
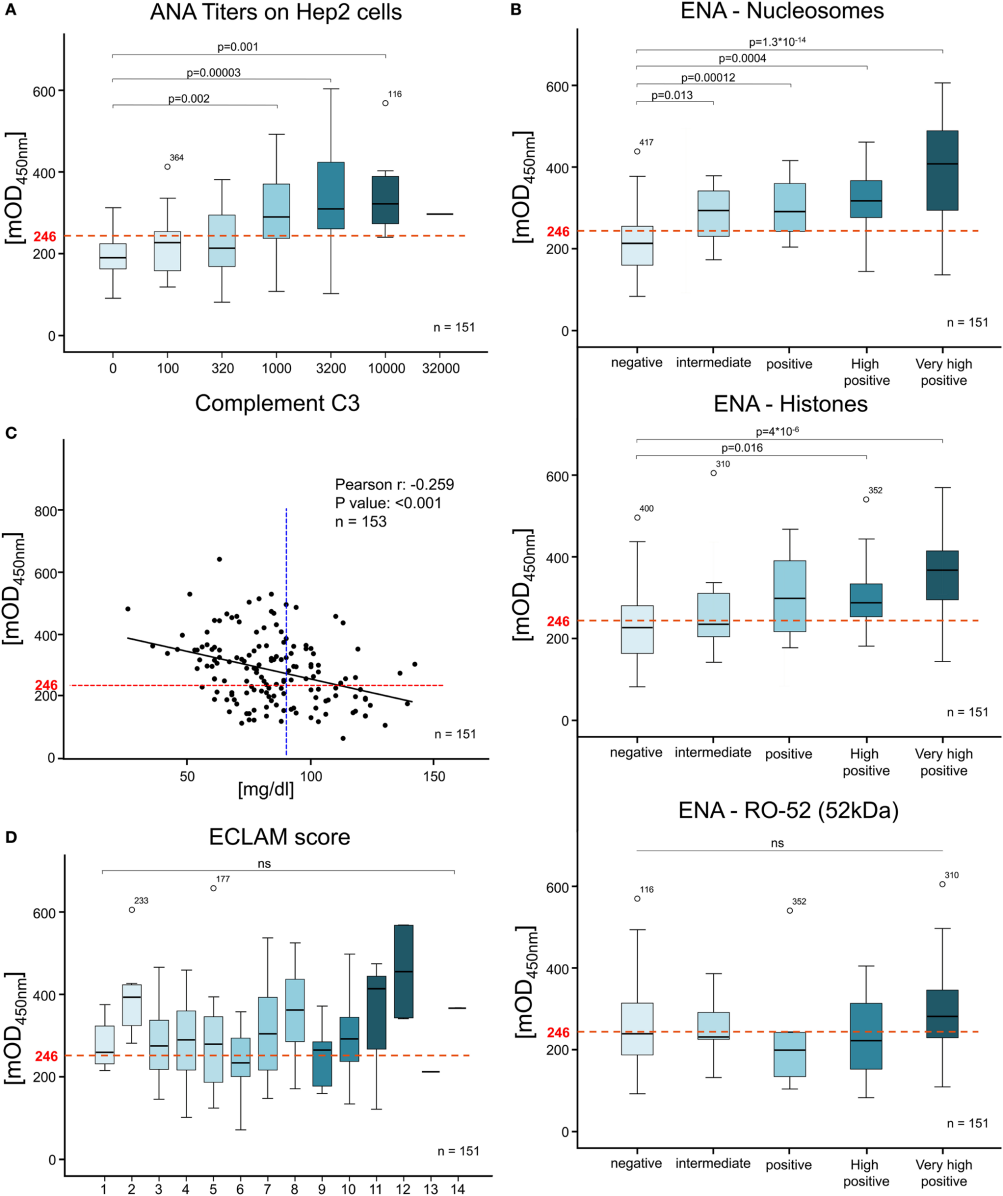
Anti-secondary NEcrotic cell (SNEC) IgG correlates with serological disease variables but not with the disease activity. The correlation of SNEC ELISA levels with serological and clinical variables was evaluated by Bravais-Pearson correlation coefficients and corrected after Bonferroni. SNEC positivity was compared to **(A)** anti-nuclear antigen on HEp-2 cell titers, **(B)** extractable nuclear antigen positivity (Nucleosomes, Histones, Ro52), **(C)** Complement C3 levels (cut off C3 levels at 90 mg/ml indicated as blue dotted line), **(D)** European Consensus Lupus Activity Measurement Manifestation Score and others (see also Tables 1, 2 and S1, S2 in Supplementary Material). The SNEC ELISA cut-off at 246 mean optical density is depicted as a red dotted line.

The most frequently affected organs in our study cohort were joints (62.1%), skin (60.1%), and kidneys (40.5%) (Table 3), a distribution similar to previous reports. None of the employed diagnostic tests [anti-nuclear antigen (ANA), *anti-dsDNA-NcX-ELISA*, RIA, and SNEC ELISA] predicted enhanced risks for any organ involvement (Figure 2C). Interestingly, the number of patients with photosensitivity or SLE-related central nervous system involvement was significantly lower if SNEC ELISA was positive (Figure 2C, red). Correlation analyzes demonstrated further associations of anti-SNEC antibodies with serological variables of SLE (Figure 3; Tables S2 and S3 in Supplementary Material).

**Table 3:** Frequencies of organ affection by autoantibody test and statistics from contingency tables.

		*n*	Frequency (%)	SNEC ELISA		Anti-dsDNA RIA		Anti-dsDNA NcX	
				χ^2^	*p*	χ^2^	*p*	χ^2^	*p*
Arthritis	Yes	95	62.1	0.03	0.853	0.15	0.697	2.58	0.108
	No	58							

Rash	Yes	92	60.1	0.02	0.893	0.06	0.805	0.10	0.751
	No	61							

Renal	Yes	62	40.5	0.37	0.545	2.18	0.140	0.70	0.401
	No	91							

Cardiovascular	Yes	43	28.1	0.03	0.859	5.64	**0.018**	1.78	0.182
	No	110							

Leukopenia	Yes	47	30.7	2.82	0.093	0.15	0.700	0.49	0.482
	No	106							

Cerebral	Yes	38	24.8	6.87	**0.009**	0.19	0.663	0.00	0.980
	No	115							

Lung	Yes	35	22.9	0.63	0.427	0.15	0.696	2.18	0.139
	No	118							

Thyroid	Yes	31	20.3	0.19	0.666	2.52	0.113	4.92	**0.027**
	No	122							

Photosensitivity	Yes	24	15.7	8.81	**0.003**	5.58	**0.018**	5.92	**0.015**
	No	129							

Vasculitis	Yes	21	13.7	0.00	0.967	0.00	0.995	0.80	0.372
	No	132							

Liver	Yes	13	8.5	3.12	0.077	0.39	0.535	3.85	0.050
	No	140							

Hemolysis	Yes	17	11.1	0.00	0.964	0.64	0.423	1.53	0.216
	No	136							

MOF	Yes	2	1.3	0.83	0.361	0.12	0.734	1.57	0.210
	No	150							


*Anti-dsDNA, anti-double stranded deoxyribonucleic acid antibodies; NcX, nucleosomes; RIA, radio immunosorbent assay; SNEC, secondary NEcrotic cells; MOF, multiple organ failure*.

*The bold fond indicates p value ≤ 0.05*.

### Anti-SNEC Antibodies Correlate With Serological Variables of SLE

In order to explore the degree of association between SNEC ELISA, *anti-dsDNA-NcX-ELISA*, RIA, and serological variables commonly used for monitoring SLE disease activity, we analyzed complement levels (C3 and C4), markers of inflammation (CRP and ESR), and levels of AAb (ANA and ENA profiles) (Figure 3; Tables S2 and S3 in Supplementary Material). One-way ANOVA analyzes revealed that the levels of anti-SNEC and *anti-dsDNA-NcX* antibodies significantly correlated with increasing titers of ANA on HEp-2 (*p* = 0.1 × 10^−26^ and 0.4 × 10^−8^, respectively) (Table S2 in Supplementary Material). Bonferroni *post hoc* testing confirmed significantly increased values of anti-SNEC antibodies for ANA titers above 1:1,000 (Figure 3A). The levels of anti-dsDNA antibodies detected by RIA did not increase proportionally with ANA titers (Table S2 in Supplementary Material). We found a significant negative correlation between complement C3 levels and all anti-nuclear antibody tests (RIA Person’s *r* = −0.223, *p* = 0.006; SNEC *r* = −0.259, *p* = 0.001; dsDNA-NcX *r* = −0.238, *p* = 0.003) (Table S2 in Supplementary Material). While CRP levels did not correlate with any of the tests, C4 negatively correlated with RIA and ESR with SNEC ELISA and anti-dsDNA-NcX ELISA (Table S3 in Supplementary Material). The anti-SNEC antibody levels correlated with 2 out of the 17 markers of the ENA profile: histone and nucleosome, both considered specific for SLE (Figure 3B). Taken together, anti-SNEC autoantibody levels significantly correlated with the most important canonical serological variables. The anti-SNEC antibody levels and the RIA assay were independent of SLE disease activity (Figure 3D; Table S3 in Supplementary Material). Approximately 50% of the patients tested showed low ECLAM values between 4 and 7 most likely due to permanent therapeutic treatment (Figure S1H in Supplementary Material).

### Phagocytosis of SNEC by Neutrophils Is Mediated by SNEC-Specific AAb

**Figure 4:**
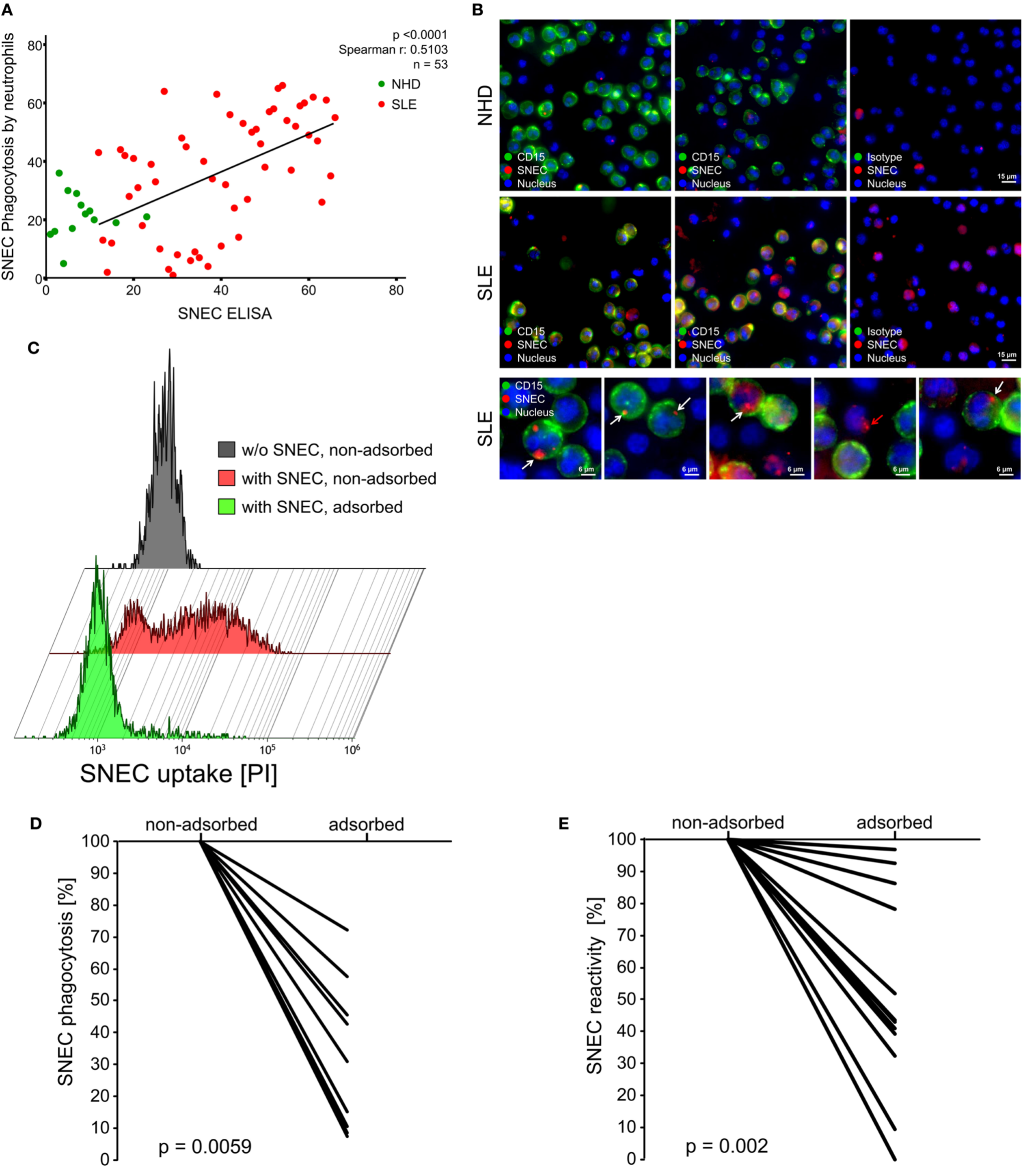
Secondary NEcrotic cell (SNEC) positivity correlates with uptake of SNEC into blood-borne neutrophils. **(A)** Correlation of SNEC uptake by neutrophils assessed by flow cytometry with anti-SNEC IgG. Values are displayed as ranks. Spearman’s rank correlation coefficient was calculated using the values of patients with SLE. **(B)** Representative microphotographs of *z*-stack series showing that PI-stained SNEC (red) was taken up by neutrophils with a segmented nucleus (CD15POS; displayed in green; Hoechst 33342POS; displayed in blue). White arrows indicate SNEC taken up by neutrophils. SNEC phagocytosed by a CD15NEG mononuclear phagocyte is indicated by a red arrow. **(C)** Uptake of PI-stained SNEC by CD16POS HLA-DRNEG granulocytes in whole blood assessed by flow cytometry in the presence (+SNEC, non-adsorbed) or absence (+SNEC, adsorbed) of anti-SNEC autoantibodies. CD16POS HLA-DRNEG granulocytes in whole blood without addition of SNEC served as negative control (−SNEC). Shown is the uptake of SNEC in the presence of a representative adsorbed and non-adsorbed SLE serum. **(D)** Percentage of SNEC uptake into CD16POSHLA-DRNEG granulocytes in the presence of 10 anti-SNEC antibody adsorbed SLE sera relative to the corresponding non-adsorbed sera. **(E)** Percentage of residual anti-SNEC IgG in adsorbed relative to the corresponding non-adsorbed sera assessed by SNEC ELISA.

We investigated the uptake of SNEC opsonized by anti-SNEC AAb (SNEC immune complexes: SNEC-IC) in a whole blood phagocytosis assay. We observed a positive correlation of uptake of fluorescent SNEC into neutrophils (CD16^POS^-HLA-DR^NEG^) in the presence of sera from SLE patients measured by flow cytometry (Spearman’s *r* = 0.5103; *p* < 0.0001) (Figure 4A). Consistent with published data, NHD with low levels of autoreactive IgG showed very low uptake of SNEC into neutrophils (Figure 4A). *Z*-stack series of immunofluorescence images confirmed the uptake of SNEC into CD15^POS^ neutrophils in the presence of a high anti-SNEC IgG SLE serum (Figure 4B). SNEC was only sporadically present in neutrophils when NHD serum was employed. Uptake of SNEC was also visible in CD15^NEG^ monocytes in the presence of both SLE and NHD sera (Figure 4B, red arrow). CD16^POS^-HLA-DR^NEG^ neutrophils displayed a substantially diminished uptake of SNEC when anti-SNEC antibodies were adsorbed from SLE serum (SNEC, adsorbed) suggesting that the uptake of SNEC by neutrophils is mainly mediated by opsonizing AAb (Figure 4C). We confirmed this result for 12 different SLE sera (Figure 4D). Despite achieving moderate (3–48%) anti-SNEC autoantibody adsorption, SNEC phagocytosis was reduced up to 85% (Figure 4E). This suggests that SNEC preferentially catches the high-affine pathologically relevant AAb from sera of patients with SLE.

## Discussion

Patients with SLE are classified based on a combination of clinical and laboratory findings. Importantly, the sole presence of biopsy-proven lupus nephritis together with positivity for ANA or anti-dsDNA antibodies is declared as sufficient for a definite diagnosis (37) emphasizing the pivotal role of circulating pathogenic AAb for classification of SLE. However, current tests employing nuclear material derived from cell lysates or recombinant proteins for the detection of SLE-specific AAb disregard the apoptotic nature of these antigens. This is particularly critical since a clearance deficiency of apoptotic cells contributes to the etiopathogenesis of SLE (6, 38).

Considering the pathophysiological events in SLE, we developed a high-throughput assay employing SNEC as target for autoreactive IgG. The major goal of our study was to establish a reproducible substrate for the sensitive detection of SLE-typical AAb. The selection of PBMCs for the production of SNEC was based on the fact that the accumulation of apoptotic material in germinal centers is the main driving force leading to the break of self-tolerance. Since lymphocytes have the capacity to proliferate and die by canonical apoptosis, we supposed that these cells are likely to mainly expose pathogenically relevant autoantigens in the context of SLE.

Our test distinguished patients with SLE from NHD with high specificity and sensitivity of 98.9 and 70.6%, respectively. In the same study cohort, *anti-dsDNA-NcX-ELISA*, and RIA, commonly used for routine testing, showed sensitivities of 43.1 or 37.9%, respectively. Although, RIA is considered the gold standard for SLE serology it does not detect low affinity AAb. Since positive test results correlate with disease severity, it is suitable for monitoring of SLE disease course and lupus nephritis in individual patients (39). Another routine diagnostic assay, the *Crithidia luciliae* immunofluorescence test, turned out to not be a reliable screening tool in unselected patients with rheumatic symptoms, although it has diagnostic value for a limited number of key SLE manifestations such as proteinuria (40). In comparison to these, SNEC ELISA appears suitable for broad, quick and cost-efficient screening of populations with presumptive diagnosis of SLE. It specifically discriminates patients with SLE from those with other rheumatic diseases such as RA, SpA, PsA, SSc, or PAPS.

Anti-dsDNA AAb do not only recognize helical B-DNA but also bent DNA present in the large intergenic regions of chromatin (41, 42). The *anti-dsDNA-NcX-ELISA* utilizes dsDNA complexed with nucleosomes for the detection of anti-nuclear antibodies. However, considering our findings, this substrate might not completely reflect the full arsenal of (apoptosis-associated) autoantigens recognized by SLE AAb. We confirmed the presence in SNEC of apoptosis-associated chromatin modifications reportedly associated with SLE (43) such as H3-K27me3, H2A/H4-AcK8,12,16, and H2B-AcK12. Consistently, standard serological variables such as ENA and ANA reactivity significantly correlated with SNEC positivity. Likewise, nucleosomes and dsDNA, representing classical autoantigenic targets for SLE-specific AAb, were accessible. We observed that HMGB1 is present in SNEC extending the repertoire of apoptosis-related autoantigens. HMBG1 reportedly to co-precipitates with anti-histone and anti-dsDNA antibodies and appears complexed with free nucleosomes in the circulation of patients with SLE (10). HMGB1-containing nucleosomes from apoptotic cells induce anti-dsDNA and anti-histone IgG-mediated responses in a toll-like receptor 2-dependent manner (44). The accessibility of HMGB1-containing nucleosomes in our assay highlights the importance of SNEC material in the pathogenesis of SLE (45).

Deposition of circulating and *in situ* formed ICs is known to be a critical factor triggering inflammation and tissue damage in SLE. They consist of assorted intracellular material exposing various modified epitopes to AAb (46, 47). Evidence that cellular debris accumulates as extracellular material in tissues exclusively in SLE patients goes back to the 50 s (48–50) and was revisited recently (51). The uptake of these ICs into phagocytes has been shown to bepro-inflammatory and mediated by FcγR (12, 18). We observed a significant correlation between the presence of anti-SNEC AAb and the uptake of SNEC into CD16^POS^ granulocytes. Some of the SLE sera employed did not facilitate phagocytosis of SNEC, although anti-SNEC IgG was measured by SNEC ELISA. Since the employed phagocytosis assay is a complex biological system, it might be less sensitive than SNEC ELISA. Thus, low amounts of AAb which can already be detected by SNEC ELISA, do not facilitate the uptake of measurable amounts of SNEC by neutrophils. Adsorption of anti-SNEC AAb from serum resulted in substantial reduction of SNEC uptake into granulocytes. Even a mild reduction of anti-SNEC autoantibody levels resulted in strongly diminished phagocytosis. Thus, we suggest that SNEC ELISA enables the detection of pathogenic AAb in SLE sera.

**Figure 5:**
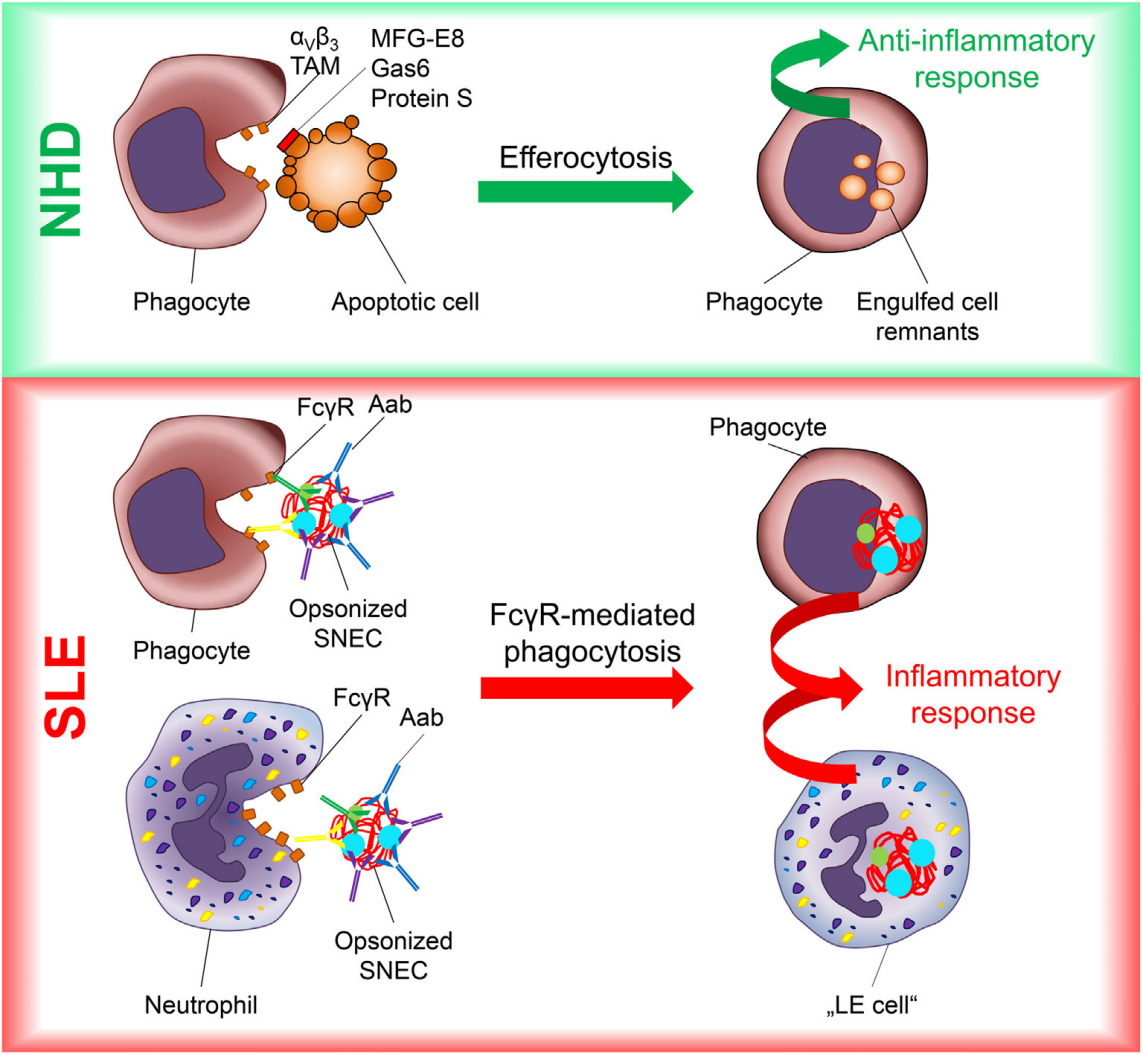
Inflammation in SLE is driven by FcγR-mediated uptake into phagocytes of secondary NEcrotic cell (SNEC) opsonized by autoantibodies (AAb). In healthy individuals (NHD), apoptotic cells are cleared rapidly and silently by professional phagocytes involving a plethora receptors and bridging molecules such as α_V_β_3_ integrin, Tryo3–Axl–Mer (TAM), milk fat globule-EGF factor 8 protein (MFG-E8), growth arrest-specific protein 6 (Gas6), and Proteins S. This marked redundancy avoids disintegration of apoptotic cells and the generation of SNEC. In patients with systemic lupus erythematosus (SLE), the concurrence of AAb and SNEC results in the opsonization and Fcγ Receptor (FcγR)-mediated uptake of SNEC into phagocytes, especially neutrophils. This perpetuates inflammatory responses and causes tissue damage. Lupus erythematosus (LE) cell.

We hypothesize that the AAb measured by SNEC ELISA induce the pathomechanism depicted in Figure 5. In healthy individuals (NHD), apoptotic cells are swiftly engulfed by phagocytes without the induction of inflammatory responses, a process referred to as efferocytosis (Figure 5) (15). The recognition of phosphatidylserine on the outer surface of the dead or dying cell is a key step in this process. It engages several different proteins and lipids including α_V_β_3_ integrin, TAM receptors (Tryo3, Axl, Mer), milk fat globule-EGF factor 8 protein (MFG-E8), growth arrest-specific protein 6 (Gas6), protein S and phosphatidylserine (15). Upon recognition, phagocytes release immunomodulatory factors including TGFβ suppressing the inflammatory responses by surrounding cells. This enables the silent clearance of billions of dead and dying cells daily. In systemic lupus erythematosus (SLE), AAb recognize nuclear material derived from apoptotic cells that had escaped canonical clearance (SNEC) (Figure 5). It is known that the formation of ICs composed of SNEC and AAb activates complement and induces inflammation *via* uptake of these opsonized complexes (12, 13, 52). Blood-borne phagocytes, especially Neutrophils, taking up opsonized SNEC have formerly been described as LE cells. In contrast to the silent efferocytosis, Fc gamma receptor (FcγR)-dependent uptake of nucleic acid containing SNEC favors the release of pro-inflammatory mediators (15, 53). It was demonstrated that FcγRIIA-mediated contact with soluble ICs results in formation of NETs *in vivo* (54). Although we did not observe release of NETs in response to anti-SNEC antibody-mediated IC uptake employing life cell imaging phagocytosis experiments, it poses an interesting topic for future investigations.

Secondary NEcrotic cell-autoantibody complexes carry DNA, which is considered a danger signal by the immune system confusing them with opsonized virus particles (*virus-mimetic*), and consequently triggers DNA sensing proinflammatory pathways. The latter include TLR-9, cyclic GMP-AMP synthase, DNA-dependent activator of IFN-regulatory factors, absent in melanoma 2, STING, gamma-interferon inducible protein-16, nucleotide-binding domain leucine-rich repeat containing protein family pyrin domain containing 3, DEAD/H-box helicase 41, and meiotic recombination 11 protein [summarized in Ref. (55)]. These molecules stimulate type I IFN expression, referred to as the IFN signature, shared by most patients with SLE, which correlates with the clinical appearance and severity of SLE (56).

This study gives insight into the mechanism of SLE etiopathogenesis with respect to autoantibody pathogenicity in terms of FcγR-mediated phagocytosis and provides the basis for improved serological screening of SLE. However, limitations of the study have to be considered. First, SLE patients were under treatment and thus often showed low disease activity. The lack of a full range ECLAM distribution might create a bias that partially explains the absence of an association of anti-nuclear antibody tests with disease activity scores. Moreover, it was demonstrated that anti-nucleosome antibodies present in serologically active but clinically quiescent patients predicted future flares (57). SNEC ELISA might detect AAb which did not result in increased disease activity yet. In fact, that the test performance is not affected by the patients’ clinical status renders SNEC ELISA a robust screening test for patients with suspected SLE. Second, our study analyzed patients from a single center. Investigations of several patient groups from different study centers (multicenter study) are required to confirm applicability to different ethnicities. The investigations of specific intracellular DNA-sensors and related signaling pathways in neutrophils will provide further insights in the pathogenesis of SLE with potential therapeutic applications. In conclusion, in this study, we confirm that SNEC uptake is intimately linked with the pathogenesis of the disease. We also demonstrate that the detection of anti-SNEC antibodies is a powerful tool supporting the diagnosis of SLE and the exclusion of other autoimmune connective tissue diseases.

## Ethics Statement

This study was carried out in accordance with the recommendations of institutional guidelines and the approval of the ethical committee of the Universitätsklinikum Erlangen. The protocol was approved by the ethical committee of the Universitätsklinikum Erlangen (permit # 54_14B). Written informed consent was given by each donor in accordance with the Declaration of Helsinki.

## Author Contributions

MB and JK planned and performed ELISA and phagocytosis experiments and conducted data analysis. SB and LM coordinated clinical data mining and performed statistical analysis. EP and JV performed monoclonal ELISA experiments and provided scientific input. JR, BH, RB, GS, and GK collected clinical data and provided scientific input. MH and LM supervised the project, planned experiments, and conducted data analysis. All authors wrote, read, and approved the manuscript.

## Conflict of Interest Statement

The authors declare that the research was conducted in the absence of any commercial or financial relationships that could be construed as a potential conflict of interest.
