# Supplementary material for: Autoantibodies Recognizing Secondary NEcrotic Cells Promote Neutrophilic Phagocytosis and Identify Patients With Systemic Lupus Erythematosus
Source: Front Immunol. 2018 May 7;9:989. doi: 10.3389/fimmu.2018.00989 (PMC5949357; doi:10.3389/fimmu.2018.00989)
Supplement: Supplementary file 1 [file Table_1.DOCX]

Supplementary Material

Autoantibodies recognizing Secondary NEcrotic Cells (SNEC) promote neutrophilic phagocytosis and identify patients with Systemic Lupus Erythematosus (SLE)

**Mona HC Biermann^1#^, Sebastian Boeltz^1#^, Elmar Pieterse^2^, Jasmin Knopf^1^, Jürgen Rech^1^, Rostyslav Bilyy^1,3^, Johan van der Vlag^2^, Angela Tincani^4^, Jörg H.W. Distler^1^, Gerhard Krönke^1^, Georg Schett^1^, Martin Herrmann^1^ & Luis E Muñoz^1*^**

***Correspondence:**

Corresponding Author: Luis E. Munoz, [luis.munoz@uk-erlangen.de](mailto:luis.munoz@uk-erlangen.de)

## Supplementary Tables

**Supplementary table 1 Summarized test performances of the analyzed tests.**

|  | **SNEC ELISA** | **RIA** | **NcX ELISA** | **RIA*ENA-Histone** | **RIA*ENA-Nucleosome** | **RIA*ENA-His*Nuc** | **ANA on Hep-2** |
| --- | --- | --- | --- | --- | --- | --- | --- |
| **Sensitivity** | 70.6 | 37.9 | 43.1 | 45.4 | 49.3 | 51.3 | 71.2 |
| **Specificity** | 98.9 | 97.6 | 98.4 | 97.4 | 94.7 | 94.9 | 76.2 |
| **Negative predictive value** | 66.2 | 30.1 | 41.2 | 30.8 | 31.9 | 33.3 | 63.6 |
| **Positive predictive value** | 99.1 | 98.3 | 98.5 | 98.6 | 97.4 | 97.5 | 82.0 |
| **Test Performance  Diagnostic odds ratio (DOR)** | 211.2 | 25.0 | 46.3 | 30.8 | 17.5 | 19.5 | 7.9 |

Abbreviations: NcX, nucleosomes; RIA, radio immunosorbent assay; SNEC; Secondary NEcrotic Cells; ENA, extractable nuclear antigens
