# Supplementary material for: Autoantibodies Recognizing Secondary NEcrotic Cells Promote Neutrophilic Phagocytosis and Identify Patients With Systemic Lupus Erythematosus
Source: Front Immunol. 2018 May 7;9:989. doi: 10.3389/fimmu.2018.00989 (PMC5949357; doi:10.3389/fimmu.2018.00989)
Supplement: Supplementary file 2 [file Table_2.DOCX]

Supplementary Material

Autoantibodies recognizing Secondary NEcrotic Cells (SNEC) promote neutrophilic phagocytosis and identify patients with Systemic Lupus Erythematosus (SLE)

**Mona HC Biermann^1#^, Sebastian Boeltz^1#^, Elmar Pieterse^2^, Jasmin Knopf^1^, Jürgen Rech^1^, Rostyslav Bilyy^1,3^, Johan van der Vlag^2^, Angela Tincani^4^, Jörg H.W. Distler^1^, Gerhard Krönke^1^, Georg Schett^1^, Martin Herrmann^1^ & Luis E Muñoz^1*^**

***Correspondence:**

Corresponding Author: Luis E. Munoz, [luis.munoz@uk-erlangen.de](mailto:luis.munoz@uk-erlangen.de)

## Supplementary Tables

**Supplementary table 2 Analysis of variance of autoantibody tests and serological variables by ANA titers.**

|  | **ANA dilution** | **n** | **Mean** | **95% CI** | **p** |
| --- | --- | --- | --- | --- | --- |
| **Anti-dsDNA RIA** | 0 | 15 | 2.1 | (0.5 to 3.8) |  |
|  | 1:100 | 29 | 8.5 | (0.8 to 16.3) | 0.082 |
|  | 1:320 | 38 | 11.9 | (5.0 to 18.8) |  |
|  | 1:1000 | 38 | 100.6 | (8.1 to 193.2) |  |
|  | 1:3200 | 22 | 23.4 | (6.2 to 40.6) |  |
|  | 1:10000 | 10 | 19.9 | (3.5 to 36.2) |  |
|  | Total | 152 | 34.7 | (11.2 to 58.1) |  |
| **Anti-SNEC ELISA** | 0 | 15 | 239.9 | (196.0 to 283.8) |  |
|  | 1:100 | 29 | 268.8 | (246.6 to 291.0) | 0.1*10^-26^ |
|  | 1:320 | 38 | 290.2 | (264.9 to 315.6) |  |
|  | 1:1000 | 38 | 354.3 | (323.1 to 385.6) |  |
|  | 1:3200 | 22 | 367.9 | (304.8 to 430.9) |  |
|  | 1:10000 | 10 | 367.5 | (300.1 to 435.0) |  |
|  | Total | 152 | 313.5 | (297.2 to 329.8) |  |
| **Anti-dsDNA NcX ELISA** | 0 | 15 | 18.2 | (2.1 to 34.3) |  |
|  | 1:100 | 29 | 59.4 | (29.2 to 89.5) | 0.4*10^-8^ |
|  | 1:320 | 38 | 68.4 | (47.1 to 89.7) |  |
|  | 1:1000 | 38 | 206.4 | (150.3 to 262.5) |  |
|  | 1:3200 | 22 | 315.3 | (189.2 to 441.5) |  |
|  | 1:10000 | 10 | 285.7 | (106.2 to 465.2) |  |
|  | Total | 152 | 146.3 | (116.1 to 176.4) |  |

Abbreviations: ANA, anti-nuclear antibodies, C3/C4, complement factor 3/ 4; CRP, C-reactive protein; dsDNA, double strand desoxyribonuclic acid; ESR, erythrocyte sedimentation rate; NcX, nucleosomes; RIA, radio immunosorbent assay; SNEC; Secondary NEcrotic Cells; CI, confidence interval.
