# Supplementary material for: Autoantibodies Recognizing Secondary NEcrotic Cells Promote Neutrophilic Phagocytosis and Identify Patients With Systemic Lupus Erythematosus
Source: Front Immunol. 2018 May 7;9:989. doi: 10.3389/fimmu.2018.00989 (PMC5949357; doi:10.3389/fimmu.2018.00989)
Supplement: Supplementary file 3 [file Table_3.DOCX]

Supplementary Material

Autoantibodies recognizing Secondary NEcrotic Cells (SNEC) promote neutrophilic phagocytosis and identify patients with Systemic Lupus Erythematosus (SLE)

**Mona HC Biermann^1#^, Sebastian Boeltz^1#^, Elmar Pieterse^2^, Jasmin Knopf^1^, Jürgen Rech^1^, Rostyslav Bilyy^1,3^, Johan van der Vlag^2^, Angela Tincani^4^, Jörg H.W. Distler^1^, Gerhard Krönke^1^, Georg Schett^1^, Martin Herrmann^1^ & Luis E Muñoz^1*^**

***Correspondence:**

Corresponding Author: Luis E. Munoz, [luis.munoz@uk-erlangen.de](mailto:luis.munoz@uk-erlangen.de)

## Supplementary Tables

**Supplementary table 3 Matrix of correlation coefficients and p values among autoantibody tests and various clinical variables.**

|  | | **a-SNEC IgG** | **dsDNA NcX** | **ANA on HEp-2** | **C3** | **C4** | **CRP** | **ESR60**  **min** | **ECLAM** |
| --- | --- | --- | --- | --- | --- | --- | --- | --- | --- |
| **Anti- dsDNA RIA** | Pearsons r | **0.203** | **0.304** | -0.002 | **-0.223** | **-0.201** | -0.08 | 0.088 | -0.090 |
|  | Significance | **0.005** | **0.000** | 0.978 | **0.006** | **0.013** | 0.341 | 0.287 | 0.267 |
|  | n | **194** | **191** | 152 | **153** | **153** | 151 | 148 | 153 |
| **Anti-SNEC ELISA** | Pearsons r |  | **0.700** | **0.271** | **-0.259** | -0.102 | -0.01 | **0.192** | 0.072 |
|  | Significance |  | **0.0001** | **0.001** | **0.001** | 0.208 | 0.936 | **0.020** | 0.379 |
|  | n |  | **204** | **152** | **153** | 153 | 151 | **148** | 153 |
| **Anti-dsDNA NcX** | Pearsons r | 00.7 |  | **0.38** | **-0.238** | -0.104 | 0.07 | **0.177** | **0.162** |
|  | Significance | 0.000 |  | **0.000** | **0.003** | 0.200 | 0.369 | **0.032** | **0.045** |
|  | n | 204 |  | **152** | **153** | 153 | 151 | **148** | **153** |

Abbreviations: ANA, anti-nuclear antibodies, C3/C4, complement factor 3/ 4; CRP, C-reactive protein; dsDNA, anti-double strand desoxyribonuclic acid antibodies; ESR, erythrocyte sedimentation rate; NcX, anti-nucleosome antibodies; RIA, radio immunosorbent assay; a-SNEC IgG, anti-Secondary NEcrotic Cell antibodies.
