# Supplementary material for: Autoantibodies Recognizing Secondary NEcrotic Cells Promote Neutrophilic Phagocytosis and Identify Patients With Systemic Lupus Erythematosus
Source: Front Immunol. 2018 May 7;9:989. doi: 10.3389/fimmu.2018.00989 (PMC5949357; doi:10.3389/fimmu.2018.00989)
Supplement: Supplementary file 4 [file Image_1.PDF]

Supplementary Material

Autoantibodies recognizing Secondary NEcrotic Cells (SNEC) promote neutrophilic phagocytosis and identify patients with Systemic Lupus Erythematosus (SLE)

Mona HC Biermann[1#], Sebastian Boeltz[1#], Elmar Pieterse[2], Jasmin Knopf[1], Jürgen Rech[1], Rostyslav Bilyy[1,3], Johan van der Vlag[2], Angela Tincani[4], Jörg H.W. Distler[1], Gerhard Krönke[1], Georg Schett[1], Martin Herrmann[1] & Luis E Muñoz[1*]

***Correspondence:**

Corresponding Author: Luis E. Munoz, luis.munoz@uk-erlangen.de

Supplementary Figures

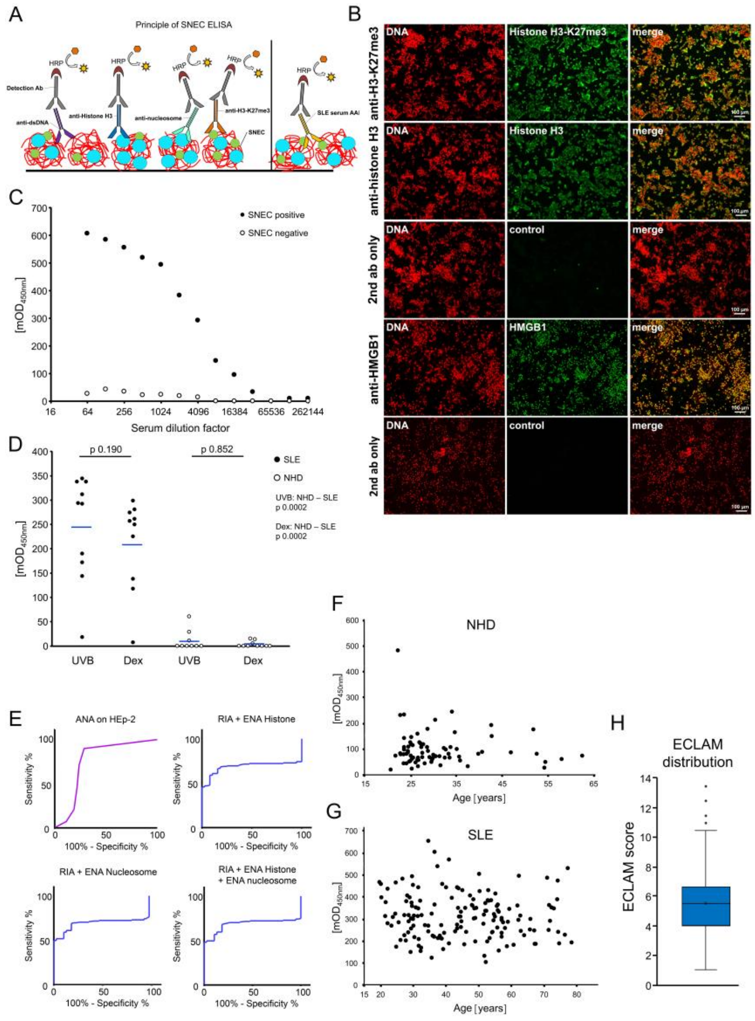

Supplementary figure 1

(A) Principle of SNEC ELISA; immobilized SNEC is recognized by autoantibodies (AAb) from the sera of patients with SLE. AAb were detected employing human IgG-specific secondary antibodies followed by a horseradish peroxidase (HRP)-mediated colorimetric reaction. (B) Immunofluorescence of histone H3, nucleosomes, and HMGB1 on immobilized SNEC counter-stained for DNA by PI; magnification 20x. (C) Serial dilution of one anti-SNEC high and one anti-SNEC low serum revealed a linear detection range from dilution factor 128 to 4000. (D) Analysis of two different apoptosis stimuli (UVB irradiation, Dexamethasone treatment) resulted in comparable autoantibody detection. Means are represented as blue lines. (E) ROC analysis of combined RIA, ENA histones and ENA nucleosomes as well as ANA on HEp-2 tests. Independency of serum anti-SNEC IgG (SNEC positivity [mOD450nm]) and age [years] was determined in (F) healthy individuals (NHD; left) und (G) patients with SLE (SLE, right). Significances and Spearman´s rho (Spearman r) for the correlation of anti-SNEC IgG positivity and age were calculated. (H) Distribution of ECLAM scores in the studied SLE cohort.
